# Supplementary material for: Topology‐Matching Design of an Influenza‐Neutralizing Spiky Nanoparticle‐Based Inhibitor with a Dual Mode of Action
Source: Angew Chem Weinheim Bergstr Ger. 2020 Jul 8;132(36):15662–6. doi: 10.1002/ange.202004832 (PMC7276915; doi:10.1002/ange.202004832)
Supplement: Supplementary file 1 — Supplementary [file ANGE-132-15662-s001.pdf]

Angewandte Chemie
Eine Zeitschrift der Gesellschaft Deutscher Chemiker

Supporting Information

Topology-Matching Design of an Influenza-Neutralizing Spiky Nanoparticle-Based Inhibitor with a Dual Mode of Action

Chuanxiong Nie, Badri Parshad, Sumati Bhatia, Chong Cheng, Marlena Stadtmüller, Alexander Oehrl, Yannic Kerkhoff, Thorsten Wolff,* and Rainer Haag**

ange_202004832_sm_miscellaneous_information.pdf

Supporting Information

Materials. All chemicals and solvents are reagent or HPLC grade, used as received, and purchased from Sigma-Aldrich (Steinheim, Germany) unless stated otherwise. The deionized water used is purified using a Millipore water purification system with minimum resistivity of 18.0 MΩ·cm. LPG with molecular weight of 10kda is synthesized in the lab as reported previously.

Synthesis of VLNP. Firstly, 0.2 g CTAB and 0.3 mL NaOH (0.1 M) are stabilized in 20 mL of water for 2 h at 70 °C with moderate stirring. Then, 20 mL TEOS, dissolved in 80 mL cyclohexane, is added into the stabilized reaction solution, and then the reaction is proceeded at 70 °C for another 72 h. The resulted VLNP is collected by centrifugation at 12,000 g for 10 min and purified by being refluxed in 100 mL acetone for 12 h. Finally, the VLNP is washed by ethanol 3 times and dried at 70 °C. The morphologies of the nanoparticles are studied by high-resolution TEM (Talos L120c, ThermoFisher, USA) after being cast on copper grids.

Synthesis of smooth NP. The smooth NP is synthesized via the conventional Stöber method. Firstly, 0.2 g CTAB and 0.5 mL NaOH (2 M) are stabilized in 100 mL water for 2 h at 70 °C. With vigorous stirring, 2 mL TEOS is added into the reaction solution quickly, and the NP is collected by centrifugation at 12, 000 g for 10 min after 2 hours. The collected nanoparticles are refluxed in 100 mL acetone for 12 h to remove CTAB and washed 3 times by ethanol and dried at 70 °C.

Synthesis of VLNP-SAL/Zan.

The VLNP is firstly amindized by APTES. Briefly speaking, 1.0 g VLNP is dispersed in 100 mL toluene and refluxed in the presence of 1.0 mL APTES at 80 ^{o}C for 2 hours. Afterwards, the VLNP-NH_2 is collected by centrifugation at 12, 000 g for 10 min and washed extensively by ethanol and water and lyophilized.

The VLNP-NH_2 is the functionalized with (1R,8S,9s)-Bicyclo[6.1.0]non-4-yn-9-ylmethyl N-succinimidyl carbonate (BCN-NHS) molecules as shown in Figure S1. 0.1 g VLNP-NH_2 is dispersed in 10 mL distilled DMF. Then, 10 mg BCN-NHS and 10 µL TEA is added into the dispersion. The solution will show a light yellow color and the reaction is processed at r. t. for 24 hours. Afterwards, the VLNP-BCN is collected by centrifugation at 12, 000 g for 10 min and washed extensively by water and lyophilized.

For the functionalization of VLNP-SAL/Zan, 0.1 g VLNP-BCN is dispersed in 10 mL distilled DMF and 10 mg LPG-SAL-N_3 (10 kDa, ~45% SAL and ~15% N_3) and 10 mg LPG-Zan-N_3 (10 kDa, ~45% Zan and ~15% N_3) are dissolved in 1mL DMF and added to the VLNP-BCN dispersion, along with 10 µL TEA. The reaction is processed at r. t. for 48 hours and the samples are collected by centrifugation at 12, 000 g for 10 min. The nano-inhibitors are washed extensively with DMF and water and frozen in PBS at -20°C for storage.

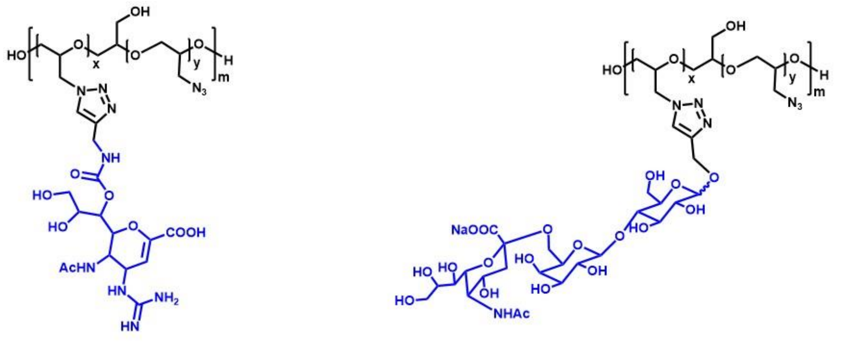

LPG-Zan-N_3
10 kda LPG, ~45% SAL and ~15% N3

LPG-SAL-N_3
10 kda LPG, ~45% SAL and ~15% N3

Figure S1. Structural information for the LPG-Zan-N_3 and LPG-SAL-N_3.

Viruses and cells. MDCK-II (Madin-Darby canine kidney epithelial) cells are maintained in monolayer cultures in DMEM (supplemented with 10% fetal calf serum, 2 mM L-glutamine, 100 mg/ml streptomycin and 100 units/ ml penicillin) at 37 °C and 5% CO_2. A/X31 (H3N2), A/PR/8/34 (H1N1) and A/Panama/2007/1999 (H3N2) are taken from the strain collection of Unit 17 at the Robert-Koch Institut (Berlin, Germany) and propagated in ten-day-old embryonated chicken eggs. The stock virus is quantitated by plaque assay on MDCK-II cells as plaque-forming unit per mL (PFU/mL).

Virus binding tests. 5 μL concentrated A/X31 (H3N2) virus solution (protein content: 0.40 mg/mL) is incubated with 15 μL 10 mg/mL nanoparticle solution for 45 min at 37 ^{o}C firstly. Then, the mixture is centrifuged at 12, 000 g for 10 mi. The unbound viral particles are removed by washing with PBS three times, and the collected nanoparticles are subjected to 10% polyacrylamide gel. After running at 200 V for 1 h, proteins are transferred onto the PVDF membrane, which is then blocked by 3 *wt*.% milk powder. The M2 protein is marked by influenza A M2 Monoclonal Antibody (Invitrogen, USA) and HRP-conjugated secondary antibody (Invitrogen, USA). Then chemiluminescent detection is performed using Pierce™ ECL Western Blotting Substrate (ThermoFisher Scientific, USA). The intensities of the bands are analyzed by ImageJ pro. By comparing the band intensity with the dose-control, the bounded viral particles are quantified.

The cryo-TEM images for the virus/inhibitor mixtures is acquired on TALOS L120c (Thermofisher, USA) after being frozen on QUANTIFOIL® R2/1 grids (Quantifoil Micro Tools, Germany).

NA activity. NA activity is investigated by 2'-(4-Methylumbelliferyl)-α-D-N-acetylneuraminic acid (MU-NANA) assay. Briefly speaking, 25 μL of virus solution (1×10^6 PFU/mL, PBS) is incubated with 25 μL nano-inhibitors in the reaction buffer (150 mM sodium acetate buffer, pH 7 and 1 mM calcium chloride) at concentrations of 25 μg/mL to 1 mg/mL for 45 min at r. t. firstly. The 50 μL MU-NANA (160 μM, in reaction buffer) is introduced to the viral/inhibitor mixture and incubated at r. t. in dark for 2 hours. The standard curve for NA activity is obtained by 2-fold dilution of the virus solution. Afterwards, the fluorescence intensity at an excitation wavelength of 365 nm and an emission wavelength of

450 nm is read for the NA activity.

Virion binding to MDCK II cells. The virions are labelled by octadecyl rhodamine B chloride according to an earlier report.[2] Then, 10 μL labelled virus (1.2 × 10^8 PFU/mL) is incubated with 90 μL nano-inhibitor solution (1.0 mg/mL) for 45 min at 37 °C. The mixture is then incubated with pre-seeded MDCK-II cells for 45 min on ice. Afterward, the cells are washed extensively by PBS, and the nucleus is marked by Hoechst 33258. The fluorescent images are acquired on SP8 lighting confocal laser scanning microscope (Leica, Germany). The virions binding to the cells are analysed by ImageJ with home-made macros. The flow cytometry is performed with similar procedure on CytoFLEX S system (Beckman Coulter, Germany) with more than 10, 000 cells being analysed.

Virus infection inhibition in single cells. For the infection inhibition test, 0.2×10^6 PFU A/X31 (H3N2) virus is treated with the nano-inhibitors for 45min at 37 °C and then used to infect MDCK-II cells which are confluent in a 24-well tissue culture plate. After 45 min of infection, the cells are washed twice by PBS and cultured in DMEM cell culture medium (DMEM, 0.1% BSA, 1100 mg/ml streptomycin, and 100 units/ml penicillin). After 24 h, the cells are washed with PBS, fixed with 2.5% formaldehyde and permeabilized with 0.5% Triton X-100. The nucleoprotein of IAV is marked by influenza A NP monoclonal antibody (Invitrogen, USA) and Alexa Fluor 594 coupled secondary antibody (Invitrogen, USA). The cell nucleus is stained by DAPI. The infection is estimated by counting infected cells from at least 10, 000 cells in total and expressed as the number of infected cells per 1, 000 cells. The inhibition ratios are estimated by comparing the infection of nano-inhibitors treated cells with the mock-infected cells as following.

$$Inhibition\ (\%) = \left(1 - \frac{Infection\ (inhibitor)}{Infection\ (control)}\right) \times 100\%$$

Multicyclic viral replication inhibition test. MDCK-II cells are firstly infected with IAV at a MOI of 0.01 and cultured in infection medium containing the nano-inhibitors for 24 h. The virus in the medium is titred by plaque assay on MDCK-II cells and are expressed as PFU/mL.

Plaque reduction assay. A/X31 (H3N2) stock is firstly diluted to 1 × 10^3 PFU/mL and then 100 μL virus solution with 100 PFU virus is incubated with 200 μL nano-inhibitors at concentration of 25 μg/mL to 1 mg/mL for 45 min at room temperature. The mixture is titrated then by plaque assay to check the active virus. The images for the plaques are acquired with a digital camera. The plaque reduction ratios are obtained by comparing the PFU for the treated samples with the non-treated virus solution as following.

$$Plaque\ reduction\ (\%) = \left(1 - \frac{PFU\ (inhibitor)}{PFU\ (control)}\right) \times 100\%$$

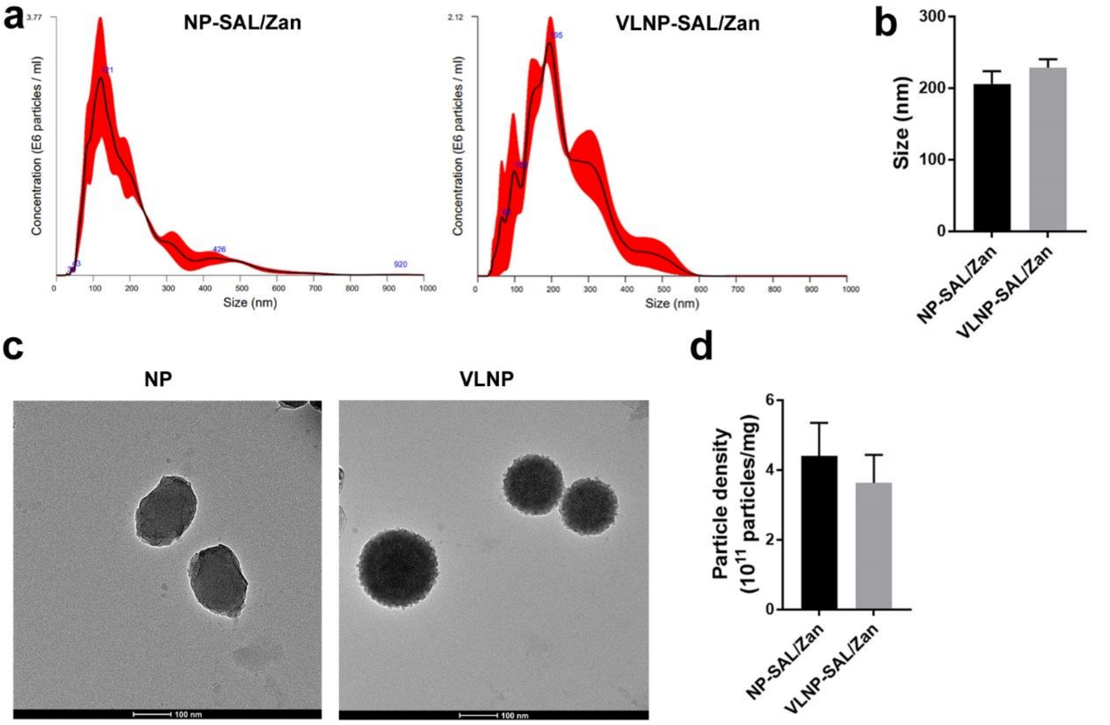

Figure S2. (a) Size distribution of the nanoparticles by NTA analysis, the results are the integration of the 3 individual measurement. (b) Diameter for the nanoparticles. (c) TEM images for smooth and rough NPs respectively. Scale bars are the same: 100 nm. (d) Particle density analysis from the NTA. Values are expressed as mean $\pm$SD, n=3. Note, the VLNP-SAL/Zan was ~10 nm larger than the NP-SAL/Zan due to the existence of the spikes on the surface, the cores were the same in size as shown in TEM images.

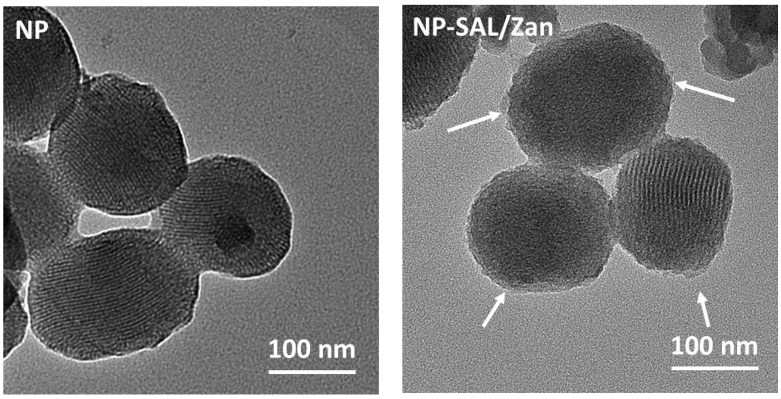

Figure S3. HR-TEM images for the LPG-SAL and LPG-Zan on the surface of smooth NP. Scale bar: 100 nm. The polymer layer on NP is marked by white arrows.

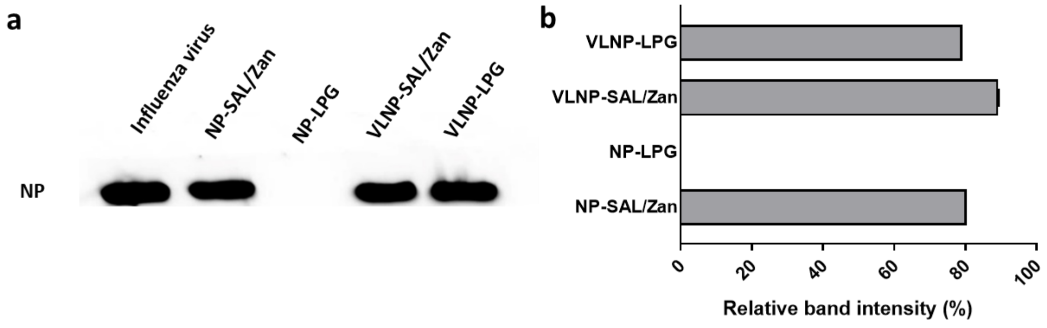

Figure S4. (a) Western blot of influenza nucleoprotein that reveals the viral binding to the nanoparticles with different morphology but the same functionalization and (b) band intensity analysis.

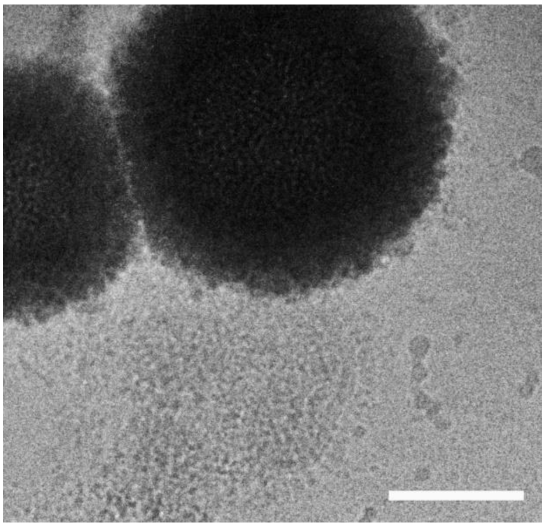

Figure S5. Cryo-EM images for the virus binding to the VLNP-SAL/Zan. Scale Bar: 100 nm.

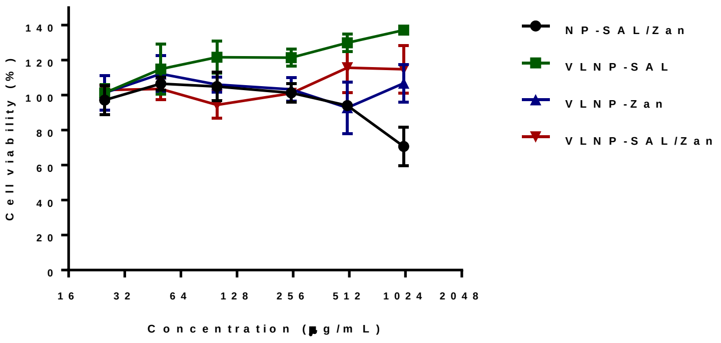

Figure S6. Dose-dependent MDCK II cell viability in the presence of the nano-inhibitors. Values are expressed as mean ±SD, n=6.

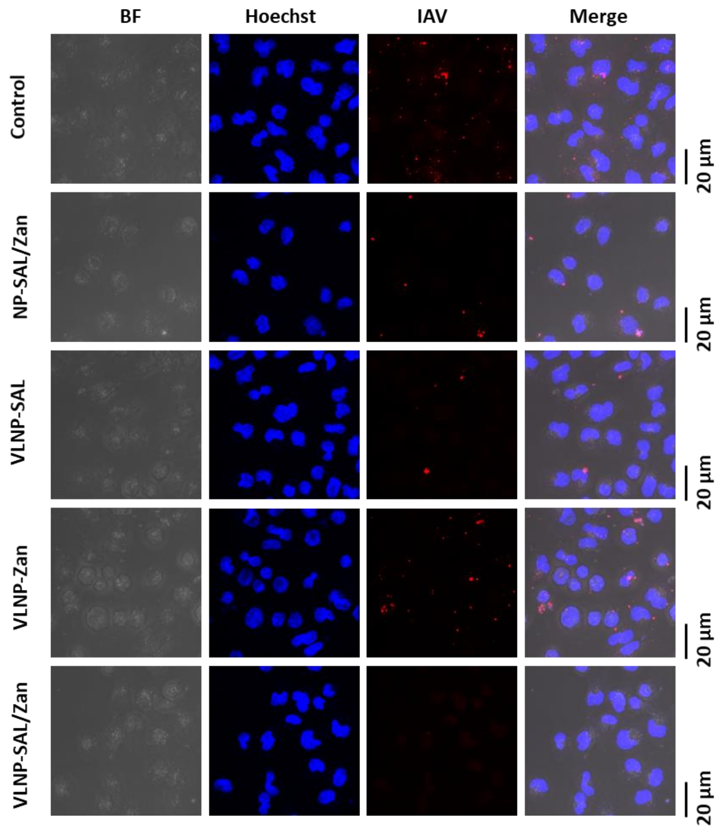

Figure S7. Detailed Z-stacked CLSM images for the IAV attachment to host cells in the presence of the nano-inhibitors. Scale bar: 20 μm.

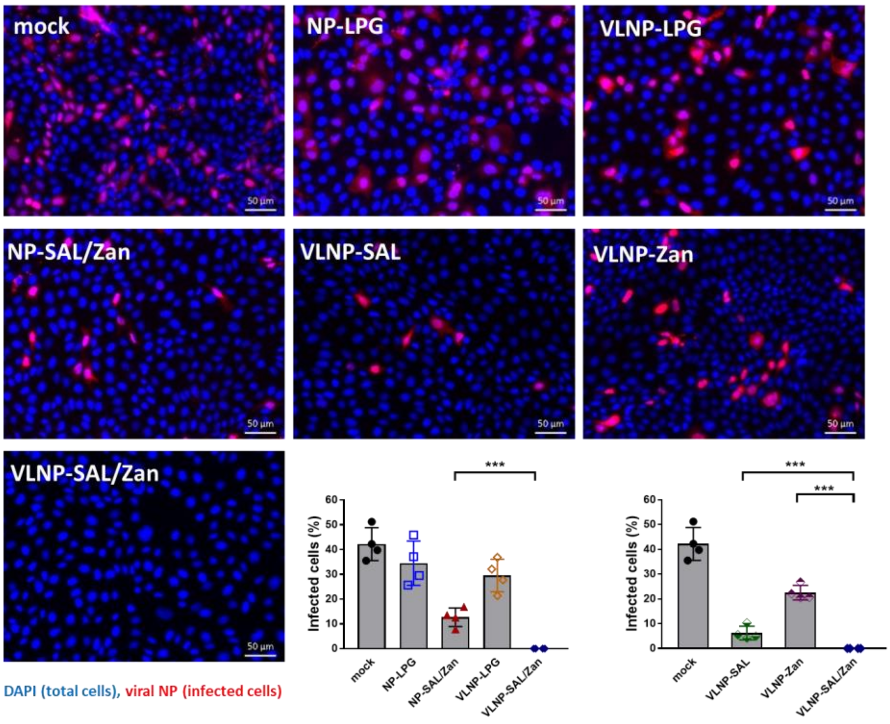

Figure S8. Detailed immune-fluorescent images for the IAV infection in single cells in

presence of the nano-inhibitors and the corresponding infected cells counting results, respectively. Scale bar: 50 μm. For the counting, more than 1, 000 cell are counted for the infection.

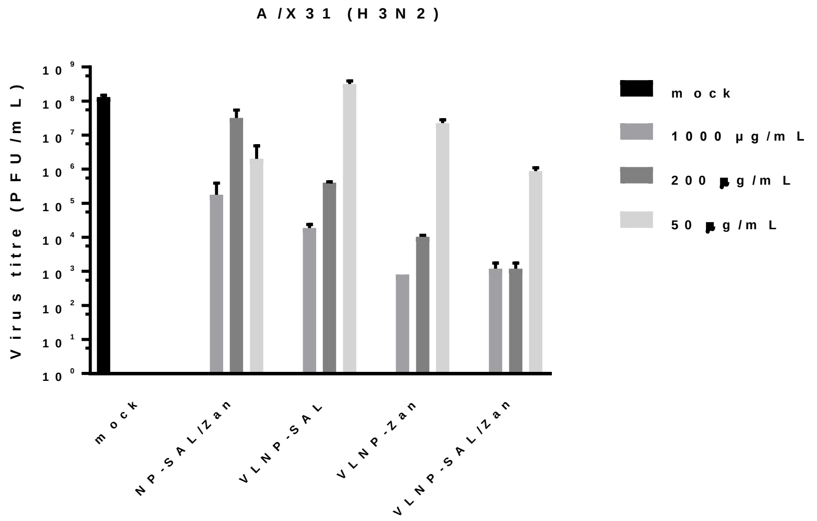

Figure S9. Late stage inhibition of A/X31 (H3N2) replication in the presence of the nano-inhibitors at different dose. Values are expressed as mean ±SD, n=6.
